# Dissection of Regulatory Networks that Are Altered in Disease via Differential Co-expression

**DOI:** 10.1371/journal.pcbi.1002955

**Published:** 2013-03-07

**Authors:** David Amar, Hershel Safer, Ron Shamir

**Affiliations:** Blavatnik School of Computer Science, Tel Aviv University, Tel Aviv, Israel; Tufts University, United States of America

## Abstract

Comparing the gene-expression profiles of sick and healthy individuals can help in understanding disease. Such differential expression analysis is a well-established way to find gene sets whose expression is altered in the disease. Recent approaches to gene-expression analysis go a step further and seek differential co-expression patterns, wherein the level of co-expression of a set of genes differs markedly between disease and control samples. Such patterns can arise from a disease-related change in the regulatory mechanism governing that set of genes, and pinpoint dysfunctional regulatory networks.

Here we present DICER, a new method for detecting differentially co-expressed gene sets using a novel probabilistic score for differential correlation. DICER goes beyond standard differential co-expression and detects pairs of modules showing differential co-expression. The expression profiles of genes within each module of the pair are correlated across all samples. The correlation between the two modules, however, differs markedly between the disease and normal samples.

We show that DICER outperforms the state of the art in terms of significance and interpretability of the detected gene sets. Moreover, the gene sets discovered by DICER manifest regulation by disease-specific microRNA families. In a case study on Alzheimer's disease, DICER dissected biological processes and protein complexes into functional subunits that are differentially co-expressed, thereby revealing inner structures in disease regulatory networks.

## Author Summary

The most fundamental and popular gene-expression experiments measure genome-wide transcription levels in two populations: perturbed and wild type, or cases and controls. The genes that show significantly different expression between the two populations (the differentially expressed genes) are useful for understanding the biology underlying the phenotype difference, and can sometimes also serve as biomarkers for classification. In contrast, genes that have similar expression to each other across all profiles (co-expressed genes) can yield clues about the functional commonality of the two populations. Differential co-expression has recently been proposed as a way to combine the benefits of these two approaches: it seeks gene groups that are co-expressed in one phenotype much more than in the other. Here we develop a new method for detecting differential co-expression and test it on case-control expression profiles of several diseases. Our algorithm improves upon the state of the art in the strength of the detected patterns and in agreement with current biological knowledge. We show that our method can predict gene regulators that are associated with the disease of interest and demonstrate that it can dissect known biological pathways into subcomponents that are not detected using standard analyses.

## Introduction

Gene expression analysis has been a central tool in biomedical research for the last two decades. Microarrays allowed genome-wide snapshots of the transcription starting from the mid-Nineties [1] and close to a million microarray profiles are available in the central databases today [2], [3]. Deep sequencing methods allow a deeper survey of the transcription, using RNA-seq [4], [5]. Fundamental methods for analysis of gene expression data use correlation between genes to infer co-expressed gene sets using clustering methods [6]–[9]. Co-expression-based methods assume that the expression patterns of the discovered gene sets are correlated in all conditions. Alternatively, biclustering methods look for gene sets that are co-expressed in a subset of the conditions [10]–[12]. Other methods compute the differential expression (DE) of a gene between two profiles or between two classes of profiles (e.g., cases and controls) [13], [14]. Co-expression analysis, biclustering and DE analysis have been highly successful in revealing gene function, and have contributed greatly to the understanding of gene regulation systems [7], [10], [13], [15]–[19].

Complex analysis of class-labeled gene-expression data has gone beyond identification of differentially expressed genes or pathways to identify differential co-expression (also called differential correlation, and abbreviated DC) patterns [20]. In DC, the co-expression of gene pairs differs between the classes. Under the premise that co-expressed genes are more likely to be co-regulated, major changes in co-expression patterns between classes may indicate changes in regulation. DC differs from clustering methods in that the discovered gene sets are not necessarily correlated across all conditions. DC methods are different from biclustering in that they make use of the known partition of the conditions into classes, and produce sets of genes with significant correlation differences between the classes, while biclusters reflect co-expression of the genes across any subset of the conditions. In addition, advanced DC methods look for pairs of gene sets with the property that the correlation between them differs between classes, and the genes in each set are correlated across all conditions. These methods provide additional information that cannot be detected by standard co-expression and biclustering methods.

Several studies have identified differentially co-expressed transcription factors (TFs) known to be involved in cancer, even though their mean expression levels had hardly changed [21]–[23]. Other studies found specific evidence for DC patterns [21]–[30] (see [20] for a review). Mentzen et al. [29] identified gene modules that are enriched with cell-adhesion- and growth-factor-related genes, and that manifest a significant decrease in co-expression in mammary gland tumors compared to wild type. In addition, several discovered gene modules were up-regulated in tumors, but had decreased co-expression within as compared to the controls. This demonstrates the complex relationship between DE and DC.

Several computational approaches have been developed for DC analysis, including detection of differentially correlated gene clusters and gene-specific analysis [31]–[34]. Simple approaches find co-expression modules using one class in the data and then test if these modules show different co-expression levels in other classes. For example, CoXpress [33] uses hierarchical clustering to find gene modules, then tests the significance of the detected modules using random sampling. Lai et al. [21] developed a statistical framework for analyzing a gene of interest, and showed that genes associated with cancer may manifest DC with many other genes. Fang et al. [35] proposed a method that looks for gene sets that are highly correlated in a large fraction of samples from one class, but in a lower fraction in the other class. Thus, the DC patterns detected are associated with a subset of the samples. Gene Set Co-expression Analysis (GSCA) [36] was proposed to test DC of known pathways. For each pathway, GSCA summarizes the change in co-expression over all gene pairs in the pathway and estimates significance using permutation tests. A recently proposed method called DiffCoEx [34] looks for DC gene modules. DiffCoEx uses a statistical framework to quantify DC. The DC measurements are transformed into dissimilarity scores between genes, and hierarchical clustering is used to detect gene modules. DiffCoEx can detect gene modules that manifest a marked change in the correlation and module-to-module changes.

**Figure 1:**
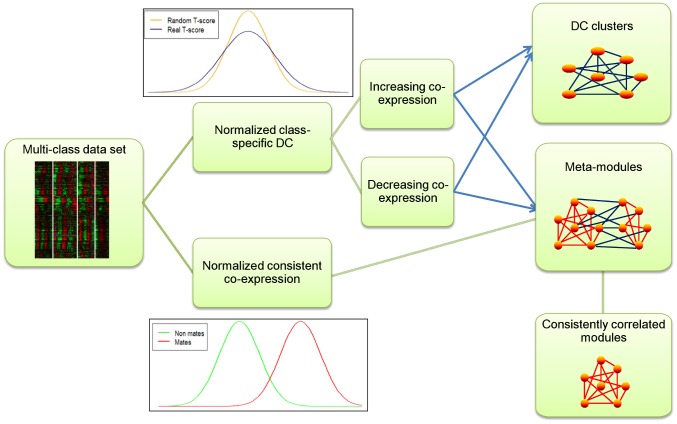
Overview of the class specific differential correlation (DC) analysis. The input (left) is a set of expression profiles from different classes of samples. In one analysis (top center), T-scores are computed for the class of interest and are normalized using the T-scores calculated on random data sets, created by shuffling the sample labels. The normalized scores are then used to find gene clusters that manifest DC in the tested class compared to all other classes (top right, up/down-correlated modules; blue edges indicate class-specific DC). A second similarity analysis (bottom center) is performed to detect gene pairs that are co-expressed in all classes. In each class, an EM algorithm is used to divide the correlations to high (‘denoted “mates,” red distribution) and low (denoted “non-mates,” green distribution), and consistent similarities are defined as cases in which gene pairs are mates in all classes. The two scores are used to find pairs of gene modules in which each module is a group of consistently correlated genes (red edges), whereas the correlation between the modules is differential (blue edges). These module pairs are denoted as meta-modules (center right). As a by-product, individual modules are recorded (bottom right).

Here we describe DICER (Differential Correlation in Expression for meta-module Recovery), a new method for DC analysis. Given a set of gene-expression profiles partitioned into classes, DICER aims to detect gene sets that manifest enhanced or reduced correlation in a class of interest as compared to each of the other classes. DICER addresses two scenarios of DC (**Figure 1**). The first is a group of genes that are differentially correlated in one class as compared to all other classes; we call such a group a *differentially correlated cluster (DC cluster)*. The second scenario refers to a pair of gene sets. The genes within each set are correlated across all the classes. The correlation between the sets, however, differs between classes. We call such a pair a *meta-module*, and each set is called a *module*. A meta-module might represent two sets of genes that are involved in a biological process, and the co-expression between the sets differs between phenotypes because the regulation of one set of genes is altered in the disease condition. DICER is freely available for download from http://acgt.cs.tau.ac.il/dicer/.

We tested the ability of DICER and extant methods to find DC gene modules in five disease-related gene-expression data sets. We discovered that DICER can detect a greater number of significant pathway enrichments, and that the modules discovered by DICER manifest stronger patterns of correlation changes. In addition, DICER modules are highly enriched with genes that are targets of miRNA families. These enrichments identify known miRNA-disease associations and suggest new candidate miRNAs that affect the tested disease. In a case study on Alzheimer's disease, we demonstrate that DICER can dissect known biological functions into biologically meaningful subunits that cannot be detected using standard DE analysis.

## Results

### Class-specific DC is prevalent

First, we tested if DC is a common phenomenon in real biological data, by analyzing five categorical disease gene-expression data sets (**Table 1**). In order to do this, we introduced a score that measures the DC of each pair of genes for the class of interest as compared to all other classes; we call this the T-score (see Materials and Methods for a more detailed description).

**Table 1:** The data sets used in this study.

Data set	Tissue	Classes	No. classes	Class of interest	No. samples	GEO ID [ref]
AD	Brain	Alzheimer's disease, controls	2	Alzheimer's disease (AD)	363	GSE15222 [62]
NDD	Brain	Six neurodegenerative diseases, controls	2	Neuro-degenerative disorders (NDD)	118	GSE26927 [n/a]
IBD	Blood	Bowel diseases (Crohn's, ulcerative colitis), controls	3	Crohn's disease	128	GSE3365 [63]
Lung cancer	Lung	Lung cancer, controls	2	Lung cancer	187	GSE4115 [64]
SLE	Blood	Inflammatory and infectious diseases, controls	6	Systemic lupus erythematosus (SLE)	270	GSE22098 [65]

**Figure 2:**
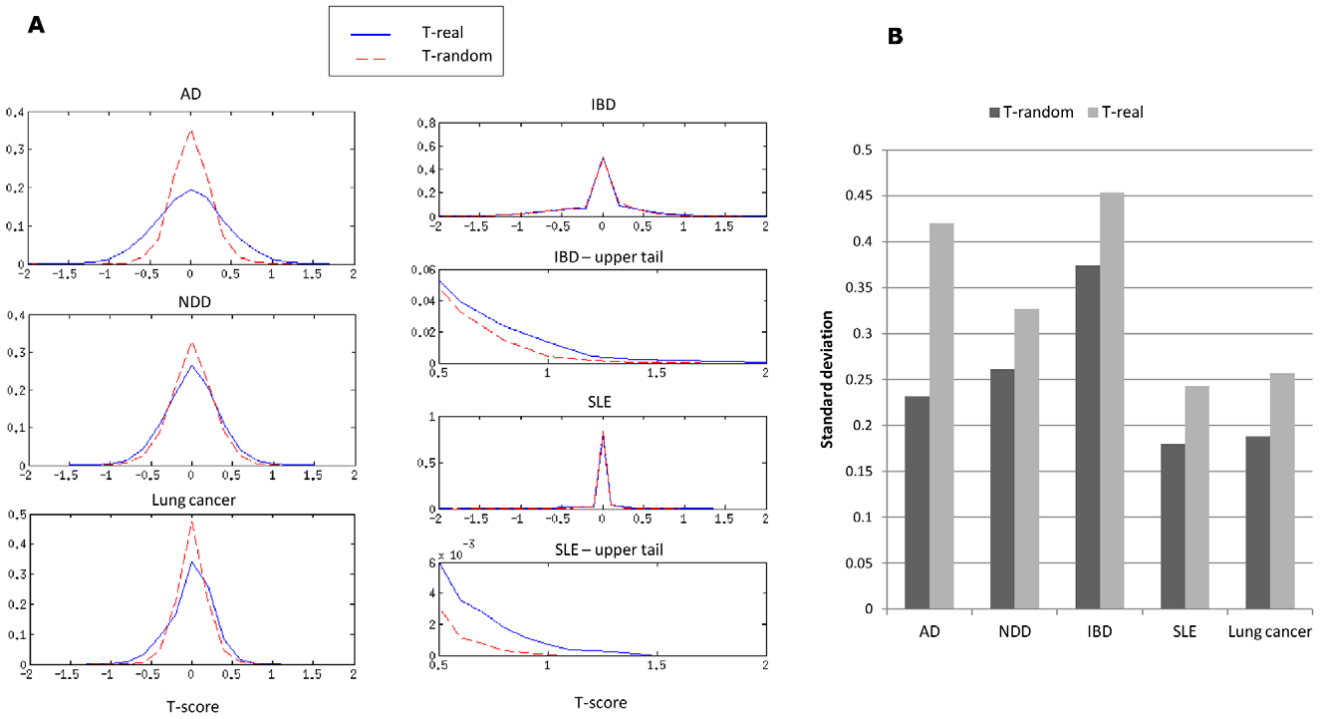
T-score distributions in real and permuted data sets. (A) The distributions of the T-scores in the real (blue) and permuted (red) data sets. The variance of the distributions is larger for the T-scores on the real data, even though the means are similar. Since in the IBD and SLE data sets most T-scores are close to zero, we also show the upper tails of their distributions. (B) The standard deviation of the T-scores in the real and permuted data sets. The standard deviation is larger in all real data sets, indicating that high T-scores (in absolute value) are more probable in the real data sets. Permuted data sets were generated by shuffling sample labels. Results are the average of 50 permutations.

For every data set, we performed random permutations of the sample labels and calculated the T-scores. If DC is prevalent, the real data sets will have higher T-scores (in absolute value) than the randomized data sets. **Figure 2a** shows the score distributions of the real and the permuted data sets. The two-class data sets (AD, NDD and lung cancer) exhibit a clear distinction between the real and permuted distributions of T-scores: both distributions are centered on zero, and the variance in the real data sets is larger. In the multiclass case (IBD, SLE), the vast majority of scores are zero for both the real and permuted data sets. The graphs that focus on the upper tails of the distributions show that the real data have heavier tails for these data sets as well. **Figure 2b** shows a comparison of the standard deviations in each data set; in all cases, the real data set has a larger standard deviation. Taken together, these results show that the real data sets have a larger fraction of extreme T-scores (high absolute values) than the permuted data sets, indicating the prevalence of DC.

### Finding phenotype-specific gene modules that are affected by DC

We developed a novel algorithm called DICER (Differential Correlation in Expression for meta module Recovery) for extracting gene modules that manifest DC with respect to a specific phenotype. DICER is freely available for download from http://acgt.cs.tau.ac.il/dicer/. The following paragraph gives a brief overview of the method, which is then described in more detail in the rest of this section. Full details can be found in the Materials and Methods section.

**Figure 1** shows an overview of the algorithm, which has three phases. First, it calculates two scores for each gene pair: one score is the DC for the gene pair between the tested class and all other classes (the T-score), and the second score identifies gene pairs that are consistently correlated across all classes. In the second phase, the DC scores are used to identify DC clusters. This analysis distinguishes between clusters with higher correlation in the target class than in all other classes, and clusters with lower correlation. In the final phase, the differentially co-expressed and consistently co-expressed gene pairs are used together to find meta-modules: a pair of modules for which the genes within each set are correlated across all the classes, but the correlation between the sets differs between classes. Because meta-module detection is NP-hard to approximate (see proof Text S1), DICER uses heuristics for this task.

The first step of DICER calculates two scores that assess the relation between gene pairs: DC score and consistent correlation score. The DC score is based on T-scores for a specific class of interest. The sample labels are randomly shuffled and permuted T-scores are calculated. The DC score is the log likelihood ratio (LLR) between the probabilities of observing the value of the pair under the distributions of T-scores for real and permuted labels. The consistency score identifies gene pairs that are consistently correlated in all classes. Our analysis follows the model presented in [9]. For each class, the correlations of the expression profiles are calculated and used to partition the gene pairs into those with high correlation (“mates”) in the class and those with low correlation (“non-mates”). Gene pairs that are highly correlated in all classes are identified.

In the second step, the DC scores are used to find DC clusters with increased or decreased correlation in the class of interest, such that the sum of DC scores in each cluster is positive (**Figure 1**, top right). The underlying model, similar to [9], ensures that the each discovered gene cluster is likely to represent a significant phenomenon. To focus on large clusters with high DC scores, only clusters that contain at least 15 genes are accepted (see Materials and Methods for further explanation).

The third and final step of the algorithm builds meta-modules (**Figure 1**, center right). A greedy procedure, akin to [37], builds one meta-module at a time by considering the DC and consistency scores of pairs that have not yet been assigned to modules. The resulting meta-modules are refined by merging meta-modules when doing so improves the overall score of a solution.

**Figure 3:**
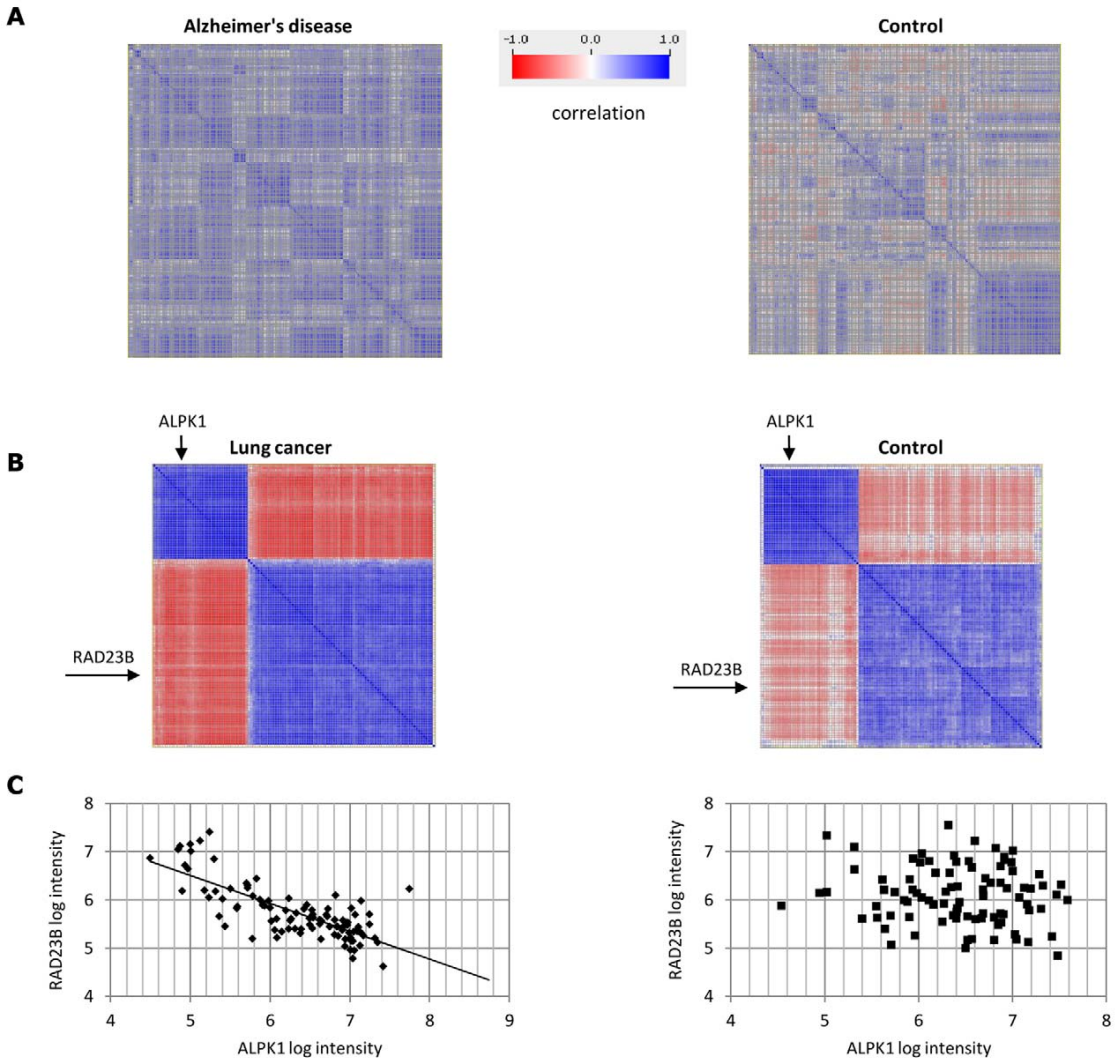
Examples of differential correlation patterns. (A) An up-correlated 242-gene cluster discovered in the AD data set. The correlation matrices of the cluster genes in the AD and control classes are shown. The average correlation is 0.72 and 0.44 in the AD and the control classes, respectively. (B) A down-correlated meta-module discovered in the lung cancer data. It contains two gene modules of sizes 39 and 77. The correlation matrices of the meta-module genes are shown for the lung cancer and the control classes. The correlation between the two modules is −0.43 in the control class, whereas the correlation in the lung cancer class drops to −0.86. Each module is a group of genes that are highly correlated in both classes: the average correlation within each module is >0.75. (C) The correlation between genes RAD23B and ALPK1 in the lung cancer data. The two genes are marked by arrows in B. Each dot corresponds to an individual and the axes mark the base-2 logarithm of expression values of the two genes in that individual. The genes are negatively correlated in the lung cancer class (r = −0.76) but are uncorrelated in the controls (r = −0.12). See Text S2 for additional examples using simulated data.

**Figure 3** shows two examples of gene sets detected by DICER in the Alzheimer's disease (AD) and lung cancer data sets. **Figure 3a** shows an up-correlated gene cluster (i.e., a cluster of genes that are more correlated in AD than in controls) of 242 genes that DICER discovered in the AD data set. The average correlation of these genes in the AD class and controls is 0.715 and 0.437, respectively. This cluster is significantly enriched with many functional terms (hypergeometric q<0.05 after FDR correction). It contains 80 genes related to cerebellum activity (p = 3.7E-10), 13 spliceosome genes (p = 1.29E-6) and genes that belong to protein complexes related to miRNA processing: large Drosha complex (6 genes, p = 7.53E-6) and DGCR8 multiprotein complex (5 genes, p = 3.1E-5). **Figure 3b** shows a down-correlated meta-module in the lung cancer data set. This meta-module contains two modules of sizes 39 and 77; the average correlation between them is −0.86 in the lung cancer class and −0.43 in the controls. The average correlation within each module is greater than 0.75 in each class. The larger module is significantly enriched with C complex spliceosome (4 genes, p = 8.4E-3) and protein complexes related to miRNA processing: large Drosha complex (3 genes, p = 4.4E-3) and DGCR8 multiprotein complex (3 genes, p = 9.7E-4). Unlike the AD case, here the miRNA-related complexes are correlated in all classes. The functional annotation for most genes in the smaller module is poor: only 2 out of 39 are assigned to GO biological processes. Thus, this meta-module suggests new candidate genes that may be related to lung cancer. **Figure 3c** shows the expression values of two genes of the meta-module, ALPK1 and RAD23B. They are negatively correlated in the lung cancer samples (r = −0.76) but are uncorrelated in the controls (r = −0.12). **Text S2** and **Figures S1, S2, S3** describes results of DICER on simulated data.

### Comparison with extant methods

In this section we compare DICER, other DC-based methods, and standard gene-expression analysis methods. We start with an explanation of the differences between DICER and extant DC-based methods, and then compare the ability of the methods to detect DC. The comparison includes extant DC-based methods, and also a clustering algorithm that uses co-expression. We show that DC-based methods are superior in detecting DC between gene sets. We then show that DICER improves upon extant DC-based methods in terms of pathway enrichment. Finally, we compare DICER, other DC-based methods, DE analysis, and co-expression analysis in terms of enrichment of miRNA targets. We shall show that of the DC-based methods, DICER is best at detecting gene modules significantly enriched with miRNA targets, and that DICER is superior to all other methods in detecting disease-specific miRNAs.

### Extant DC-based methods

Extant DC-based methods look for gene modules with altered correlation patterns between classes. The CoXpress method [33] uses hierarchical clustering on the expression patterns of one class to find gene modules. DiffCoEx [34] hierarchically clusters the genes after transforming the correlation differences into distances. Unlike DICER, DiffCoEx outputs modules and not meta-modules. DiffCoEx then looks for “module-to-module” relation: a pair of modules with high DC between them. This is equivalent to a meta-module, but without requiring high correlation within each module. Pairs of DiffCoEx modules can be tested to find those that are meta-modules. Another important difference is that DICER uses statistical normalization of the DC scores to ensure that the accepted modules are significant.

Unlike GSCA [3], which searches for DC patterns among known pathways, DICER does not use any prior information. Therefore, analysis of the meta-modules found by DICER can reveal differential relations between genes of different biological processes. Moreover, such analysis can dissect the genes of a single biological process into subgroups that are differentially correlated, as we shall show in a case study on AD data.

### Extent of DC between gene modules

We applied DICER, CoXpress and DiffCoEx on the five data sets described in **Table 1**.The full list of gene sets found by DICER is available in **Text S3**. In all cases, CoXpress found no significant clusters that contain at least 15 genes. DiffCoEx and DICER detected modules in all data sets. On average, the DiffCoEx solution contained 4.2 meta-modules that covered 29% of the genes, whereas DICER found an average of 23 meta-modules that covered 34% of the genes (see **Table S1** for module statistics). DICER detected DC gene clusters with at least 15 genes only in the AD and SLE data sets.

**Figure 4:**
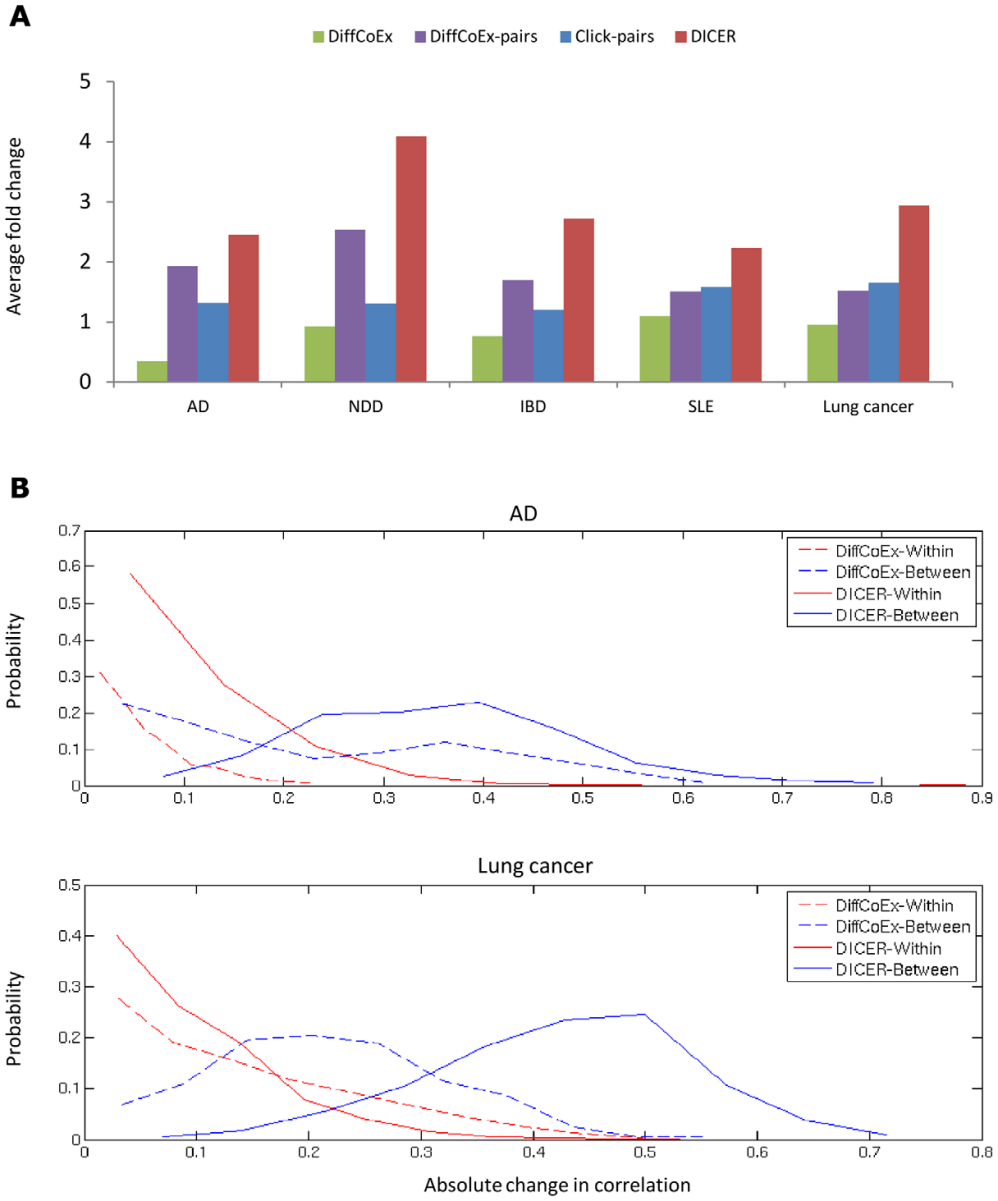
Comparison of absolute difference in correlations in gene sets found by different algorithms. (A) The extent of DC compared to random gene sets. For each discovered module and module pair we created 200 random gene sets of the same size and calculated their absolute DC. We then calculated the ratio between the scores of the discovered modules and the mean of the random gene sets. The green bars show the mean of the top two DiffCoEx modules in each data set. For testing DiffCoEx and CLICK module pairs (purple and blue bars respectively), we took into account only module pairs with fold change greater than 1.1. CoXpress found no significant clusters of 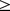
15 genes. For DICER (red bars), the top ten up-correlated and the top ten down-correlated module pairs were taken into account. (B) The distribution of within- and between-module absolute change in correlation for DICER and DiffCoEx in the AD and lung cancer data sets.

We compared DICER, DiffCoEx, and the CLICK unsupervised clustering algorithm [9] in terms of the extent of DC between modules. We started by testing the extent to which each method gave nonrandom results. For each method and each pair of modules, we created 200 random module pairs by randomly selecting gene sets of the same sizes. The random modules had the same numbers of genes as the pair of modules being tested. We calculated the absolute change in correlation between each pair of modules, and measured the ratio of the change between the real module pair and the mean of the random module pairs. A similar procedure was applied to each DiffCoEx module. Many DiffCoEx modules and module pairs did not manifest a ratio greater than 1. In order to make the comparison more meaningful, we included only the DiffCoEx modules with the two highest ratios in each data set. We also included only DiffCoEx and CLICK module pairs with ratio greater than 1.1. The results are shown in **Figure 4A**. For all data sets, the ratio computed between DiffCoEx module pairs was higher than the ratio within modules, and the ratio within DiffCoEx modules was close to or smaller than 1. The ratios for DICER meta-modules were the highest in each data set. Thus, both DiffCoEx and DICER can discover differentially co-expressed module pairs, but those found by DICER exhibit stronger signals. We also inspected the within- and between-module absolute change in correlation. Results on the AD and lung cancer data sets are shown in **Figure 4B**. Both DiffCoEx and DICER achieve marked separation between the within- and between-module correlation changes. The distribution of between-module correlation changes of DICER modules is significantly shifted towards higher values compared to DiffCoEx (Kolmogorov-Smirnov p<1E-20).

### KEGG pathway enrichment analysis

**Figure 5:**
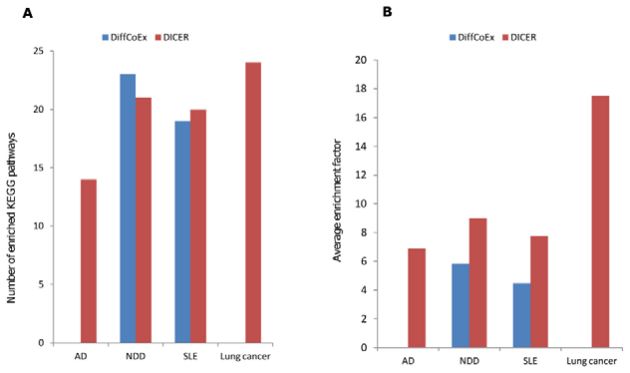
KEGG pathway enrichment analysis. The modules found by DiffCoEx and DICER were tested for KEGG pathway enrichment using the hypergeometric test with 0.05 FDR correction. Neither method reported significant enrichment on the IBD data set. (A) The number of enriched pathways. (B) Average enrichment factors of the enriched sets. The enrichment factor is the ratio between the fraction of the pathway genes in the tested set and the fraction of the pathway genes in the data set.

We compared the functional enrichment of DICER and DiffCoEx modules by performing KEGG pathway enrichment analysis (hyper-geometric q<0.05, see Materials and Methods). The enrichment factor of a pathway in a module is the ratio between the fraction of the pathway genes in the tested set and the fraction of the pathway genes in the data set. The results are shown in **Figure 5A**. Neither method achieved significant enrichment on the IBD data set. In the AD and lung cancer data sets, DiffCoEx did not achieve significant enrichment, while DICER gave 14 and 24 enriched pathways, respectively. In the NDD and SLE data sets, both methods reported similar numbers of pathways. In addition, for the pathways counted in **Figure 5A**, we calculated the pathway-enrichment factor for each method on each data set. The mean of the enrichment factors found by each method are reported in **Figure 5B**. In all cases, DICER had an average enrichment factor that is at least 50% higher than DiffCoEx.

### microRNA analysis

Since co-expression may result from co-regulation, changes in co-expression may be the result of changes in regulatory patterns. Therefore, we tested if gene sets of different kinds, modules (i.e., parts of discovered meta-modules), meta-modules, or DC clusters, are significantly enriched with genes that are targets of specific miRNA families. We used the FAME algorithm for miRNA-target enrichment analysis [38]. The significance threshold was set to q = 0.05. Except for the modules in the lung cancer data, for every combination of data set and gene set type, DICER revealed at least two significant miRNAs. The different gene set types provide complementary insights. The results are shown in **Figure S4** (see **Tables S2, S3, S4, S5, S6** for the full list of families in each case). Notably, some miRNA families were found in multiple gene sets. For example, miRNA family mir-124/506 was detected in two meta-modules in the AD data set. In each data set, some miRNA families were detected only in the meta-modules or only in the gene clusters. In contrast, DiffCoEx obtained very few significant miRNA enrichment results: none in IBD, SLE and lung cancer, and one each in AD and NDD. Standard DE analysis (i.e., using a t-test with q<0.05 for identifying up- and down-regulated genes) and the CLICK algorithm achieved a comparable number of miRNA enrichments to DICER. CLICK achieved at least 15 enrichments in each data set, while the t-test found two and three enrichments in the lung cancer and NDD data sets respectively, no enrichments in the SLE data, and more than 15 enrichments in the AD and IBD data sets.

To test if the discovered miRNA families are known to be associated with the disease, we used the mir2disease database [39] and tested the significance of the overlap between the detected miRNA families and the annotations in mir2disease (see Materials and Methods). Mir2disease contains miRNA-disease associations for AD, NDD, and lung cancer. In AD, significant overlaps were obtained with clusters (six known AD-related miRNA families, p = 0.0023), and borderline overlaps were detected with modules (six miRNA families, p = 0.068). In NDD, significant overlap was obtained with modules (all five miRNAs detected in the modules are associated with NDD, p = 0.0016). In lung cancer, seven of twelve enriched miRNA families detected in meta-modules are associated with the disease (p = 0.037). DiffCoEx obtained no significant enrichment with any of these diseases. Overall, DICER detected 18 correct miRNA-disease associations. The CLICK algorithm discovered only five, and DiffCoEx and t-test of DE discovered only two. We conclude that DICER is much better at detecting disease-specific miRNAs, both in terms of the number of miRNAs and in terms of the significance of miRNA target enrichment.

### Case study: Alzheimer's disease

Neurodegenerative disorders are characterized by a progressive loss of neurons. Excitotoxicity and apoptosis are two main causes of neuronal death [40], and related pathways such as oxidative stress and mitochondria impairment have been shown to play a key role in these processes [41]. For example, many apoptotic signals emerge from mitochondria [42]. The specific causes of most of these disorders are still unknown [43]. Alzheimer's disease (AD) is the most common progressive neurodegenerative brain disorder in humans. AD is a complex progressive condition that involves sequentially interacting pathological cascades, including the interaction of amyloid-β (Aβ, APP gene) aggregation with plaque development, and the hyperphosphorylation and aggregation of tau protein as well as the formation of tangles. Together with associated processes, such as inflammation and oxidative stress, these pathological cascades contribute to loss of synaptic integrity and progressive neurodegeneration [44].

We compared the enriched miRNA families that were detected by DICER to the mir2disease database. These miRNA families cover 10 (24%) of the miRNAs that are known to be associated with AD: mir-101, mir-106, mir-124a, mir-125, mir-26b, mir-29a, mir-29b-1, mir-363, mir-9, and mir-93. Furthermore, three of these miRNAs are annotated as causal for AD: mir-106, mir29a, and mir-29b-1. The FAME analysis of the DICER meta-modules suggests additional candidate miRNAs that are relevant to AD. For example, mir-216, which was enriched in an up-correlated meta-module (q = 0.001), is predicted by Targetscan [45] to target the solute-carrier gene SLC1A2, which is important in excitatory glutamate clearance in the central nervous system. This miRNA was shown to be expressed in glioblastoma and astroblastoma cell lines [46]. Together with mir-203, which was enriched in an up-correlated cluster (q = 5E-4), mir-216 was validated as targeting the GABA receptor α1 subunit [47]. GABA receptors are known as the inhibitory receptors in the central nervous system [48]. Taken together, this shows that DICER detects well-established disease-related regulatory factors, and can also point out new candidates that may affect the disease.

**Figure 6:**
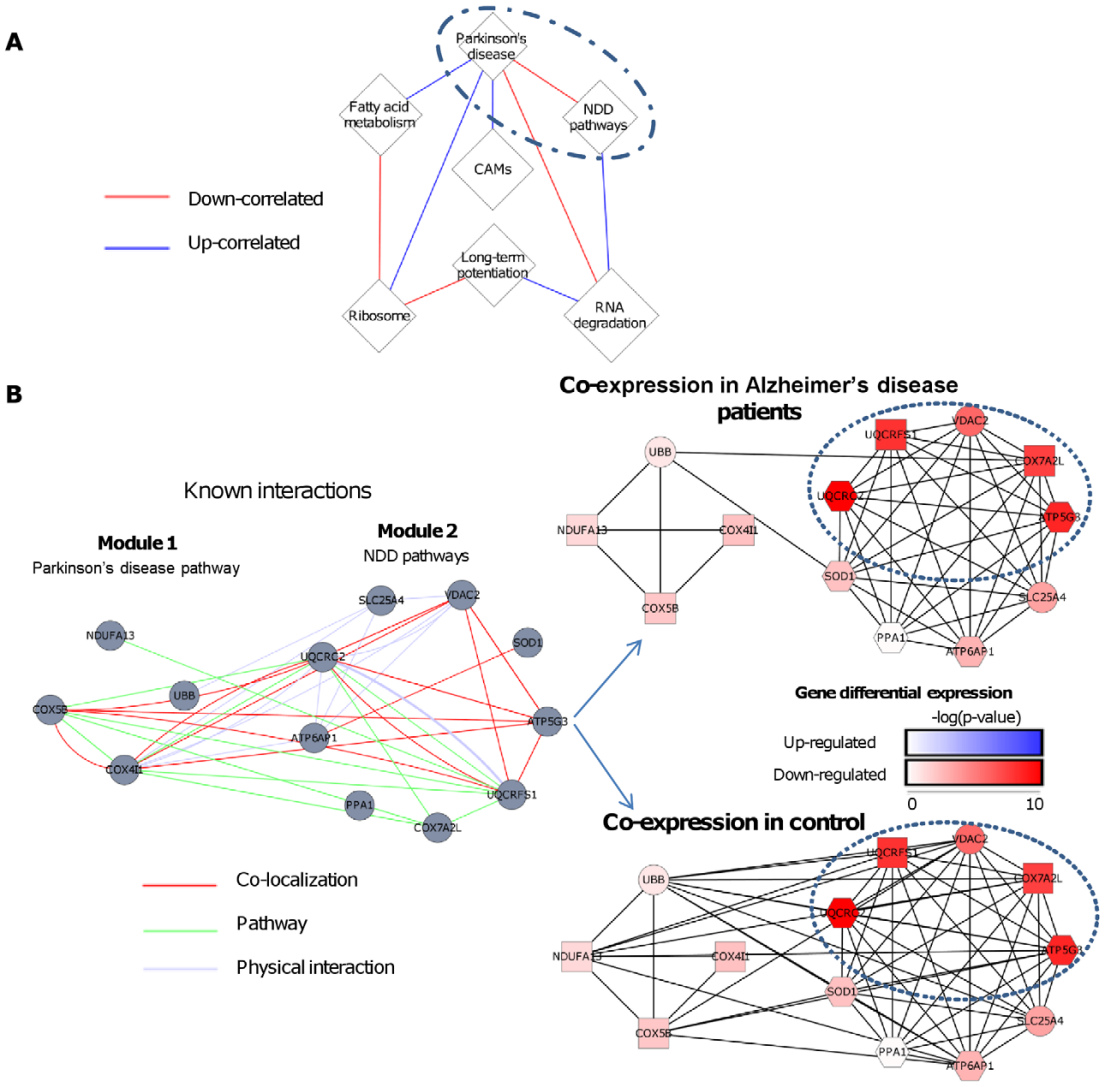
DC map of modules enriched with KEGG pathways discovered in the Alzheimer's disease data. (A) DC map of modules enriched with KEGG pathways. Nodes represent gene modules and edges correspond to DC (blue for increased correlation in AD, red for decreased correlation). Node size is proportional to the size of the module. The enriched pathways are noted on the module. NDD pathways refer to Parkinson's disease (PD), Huntington's disease, Alzheimer's disease and oxidative phosphorylation. CAMs refer to the cell adhesion molecules pathway. (B) Analysis of DC between the PD and the NDD modules (the circled sub-graph in A). Left: the known interactions involving the genes of the two modules according to GENEMANIA. Most known interactions are between the modules. Right: co-expression networks of the same genes for AD patients and controls. Rectangular nodes are genes related to oxidoreductase activity, hexagons indicate genes related to phosphate metabolic process. An edge between two genes indicates correlation >0.3 in the tested class. The average correlation between the modules was 0.3 in the controls and 0 in the AD class. Node colors indicate the DE between case and control, measured by the base-10 logarithm of the p-value (t-test) of the tested gene. The genes circled in the NDD pathway module are also part of the PD pathway. These genes are also down-correlated in AD, whereas all other genes show only mild DE.

**Figure 6A** shows a DC map of DICER modules that are enriched with KEGG pathways (q<0.05 followed by redundancy filter, see Materials and Methods). For example, a module enriched with cell adhesion molecules (CAMs) was found to be up-correlated with a module enriched with genes related to Parkinson's disease (PD). The latter module was down-correlated with a module enriched with pathways that are directly related to NDD (oxidative phosphorylation, PD, AD, and HD). **Figure 6B** shows the meta-module of PD (module 1) and NDD (module 2) in detail. GENEMANIA analysis [49]–[51] reveals that known interactions are found mainly between the modules (co-expression and predicted interactions were excluded). Although the genes in the two modules share similar functionality, the DICER analysis identified a meta-module structure, in which each module is highly homogeneous (correlation above 0.7 in both cases and controls), whereas the correlation between them is lower in AD samples (correlation of 0 in AD and 0.23 in controls; class-specific co-expression networks are shown). Both groups contain genes related to PD (all genes in module 1, and the circled genes in module 2), oxidoreductase activity (rectangular nodes: module 1 contains COX4l1 COX5B, and NDUFA13; module 2 contains COX7A2L, SOD1, and UQCRFS1), and apoptosis (NDUFA13 in module 1 and VDAC2 in module 2 [52]). Decreased co-expression within the oxidative phosphorylation process may be associated with mitochondrial dysfunction, which is well established in neurodegenerative disorders [53]. Specifically, it was shown that nuclear-encoded COX subunits 4 and 5B fail to enter the mitochondria in AD [54], [55]. DICER points out to a distinct role of these two subunits by separating them from the other COX genes and placing them on the other side of the meta-module. In addition, only module 2 contains genes that are directly related to phosphate metabolic process (hexagonal nodes: genes UQCRC2, ATP6AP1, SOD1, ATP5G3, and PPA1 This example demonstrates that disease-specific DICER analysis can detect substructures corresponding to distinct functionality within pathways without using any prior knowledge.

**Figure 7:**
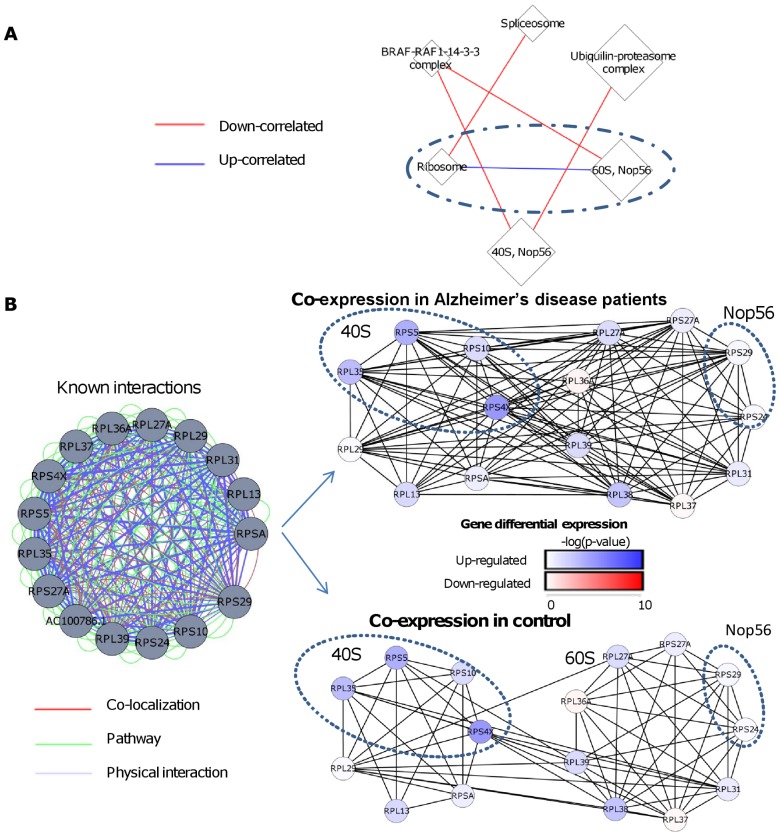
Ribosomal sub-complexes discovered in the Alzheimer's disease (AD) data. (A) A DC map of modules enriched with protein complexes. Node size is proportional to the size of the module. The enriched pathway names are noted on the module. 40S: 40S cytoplasmatic Ribosome complex, 60S: 60S cytoplasmatic Ribosome complex, Nop56: Nop56p-associated pre-rRNA complex. Blue and red edges mark increased and decreased correlation in AD, respectively. (B) Analysis of DC in the Ribosome and 60S-Nop56 meta-module circled in A. Left: the known interactions involving the genes of the two modules according to GENEMANIA. Right: co-expression networks of the same genes for AD patients and controls. An edge between two genes indicates correlation >0.5 in the tested class. The average correlation between modules was 0.4 and 0.75 in the controls and AD class, respectively. Node colors show DE between AD and control, measured as the base-10 logarithm of the p-value (t-test) of the tested gene. Circled subgroups: proteins belonging to 40S cytoplasmatic Ribosome and Nop56 complex. 40S complex genes are up-regulated in AD, whereas 60S genes show only mild DE.

**Figure 7A** shows the DC map of modules enriched with protein complexes (q<0.05) in the AD data. Here again DICER detects DC between different protein complexes, for example, decreased correlation between the spliceosome and ribosome genes. In **Figure 7A**, two up-correlated gene modules that are enriched with ribosomal genes are marked. The first module is enriched with cytoplasmatic ribosomal genes (seven genes, p = 0.002), whereas the second is enriched with both 60S ribosomal unit genes (seven genes, p = 1.68E-7, enrichment factor 21.3) and genes that belong to the Nop56p-associated pre-rRNA complex (six genes, p = 5.09E-6, enrichment factor 12.2). We note that these two complexes overlap: four of the six Nop56 genes are annotated as members of the 60S complex. In addition, only the first module contains 40S ribosomal unit genes (four genes). **Figure 7B** focuses on these two modules. GENEMANIA analysis of the modules indicates that all ribosomal genes, from both modules, are highly connected in all three types of interactions: co-localization, physical interaction, and pathway (co-expression and predicted interactions were excluded). However, by comparing the co-expression in controls and AD we observe that the modules are highly correlated only in AD. In addition, 40S complex genes are up-regulated in AD, whereas 60S genes show only mild DE. Thus, we observe increased coordination of the ribosome subcomplexes and increased activity of 40S, indicating major transcriptomic changes in AD.

## Discussion

Differential co-expression (DC) analysis can provide complementary information to standard differential expression (DE) analyses. In this study we aimed to learn meaningful DC patterns without using external biological information as part of the pattern recognition process. We developed a method called DICER, which extracts differential gene clusters and meta-modules by analyzing only a labeled gene-expression matrix. Only after this process do we use additional data to understand the biological significance of the discovered gene groups.

In this study we developed a new statistical measure of DC, which can be naturally incorporated in the analysis of a class of interest in multiclass data sets. We have demonstrated that the new measure is more likely to achieve high scores in real biological data sets as compared to shuffled data sets. This has two consequences: first, it allows us to investigate if DC is abundant in biological systems; second, it motivates normalizing the scores of real biological data sets according to the distribution of the shuffled data, using the log-likelihood ratio score. DICER's final score for a gene pair is positive only if the DC of the gene pair is high and is therefore more likely to represent a real change. This score can be naturally incorporated in graph algorithms that detect gene modules.

We compared DICER to two state-of-the-art algorithms: CoXpress, which looks for DC clusters, and DiffCoEx, which can detect both DC clusters and module-to-module relations (i.e., meta-modules). In our experiments we observed that DC occurs more often between modules than within clusters. That is, in most cases, none of the tested algorithms found DC clusters (a group of genes that are collectively more–or less—correlated in the class of interest compared to the other classes). In contrast, in all data sets, both DICER and DiffCoEx detected meta-modules with significant co-expression changes between the modules. As our tests indicate, DICER is more sensitive.

Under the premise that co-expressed genes are likely to be co-regulated, we speculated that major changes in co-expression may be caused by gene regulators with altered activity in the disease of interest. To test this, we adopted a reverse-engineering paradigm in which we identified miRNA families whose targets are enriched in the gene groups detected by DICER, and tested if these miRNA families are associated with the relevant diseases. In all the experiments, targets of miRNAs associated with the disease were significantly over-represented in the gene groups. Furthermore, we discovered that in all three diseases for which associated miRNAs were reported in the mir2disease database, the identified miRNAs were significantly enriched. Thus, we demonstrated that DICER analysis can be used to pick out disease-specific miRNA families.

Using standard functional enrichment tests on the modules detected by DICER, we were able to draw a “meta-graph” of functional terms, in which edges represent DC between biological entities. We performed this analysis using two types of annotations: biological processes and protein complexes. In most cases, DC is observed between different pathways or protein complexes. In specific cases, however, DC is found between genes in the same biological group; this represents DC between subcomponents of a biological process. We focused on two such cases in Alzheimer's disease (AD) and showed that the subcomponents detected by DICER are consistent with previous biological knowledge. First, DICER found two groups of ribosomal genes, one enriched with 40S genes and the other with 60S genes, even though DICER did not use any prior knowledge about these categories. We showed that all ribosomal genes are correlated in AD, whereas the correlation between the 40S and 60S genes is significantly lower in control samples. Second, DICER dissected NDD related pathways into subunits that show DC patterns in AD. Notably, these subunits were identified in both cases based solely on expression data.

DICER also calculates the consistency in correlation of gene pairs. Although not the focus of this study, we note that this score can be used to extract gene modules that are highly correlated across different classes. Many graph clustering algorithms can do this as well; DICER, however, normalizes the co-expression measurements of each class separately, and so can find modules from different data sets in an integrated manner.

The statistical assumptions assumed by our model are quite strong, and may not hold for all data sets. However, they provide a theoretical basis on which rigorous scores can be computed, and their utility is eventually tested experimentally. Moreover, DICER can be generalized to handle relaxed assumptions, e.g., by replacing the normal distribution by other theoretical or empirical distributions.

In summary, we presented a threefold contribution to the analysis of gene-expression data. First, we developed a statistical measure of DC and consistent correlation across different classes. Our statistical analyses demonstrated that DC is abundant in real biological data sets. Second, we developed an algorithm to detect DC clusters and meta-modules. We showed that our algorithm improves upon state-of-the-art algorithms. Finally, we demonstrated how new biological insights can be obtained using our analysis. This comprised discovering disease-specific miRNA families and dissecting biological process into functional subcomponents with disease-specific co-expression.

## Materials and Methods

In this section we provide the statistical basis for and algorithmic description of DICER. We first define a new DC score and a likelihood framework for using it. We then describe how to perform class-specific DC analysis and how to find DC clusters and meta-modules.

### A normalized score for DC

We first define a new normalized score for DC; it will be the basis of our analysis. For genes u,v and a class D of profiles, define 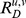 to be the Pearson correlation between u and v in class D. Given two classes 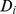 and 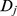 and a pair of genes u and v, we assume that the correlations within each class are normally distributed with class-specific parameters:
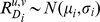


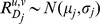
We also assume that the correlations are independent. Hence, the expected distribution of the difference satisfies:
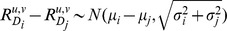
These assumptions are very strong and do not necessarily hold for our data. In particular, dependencies of correlations are expected, since the same gene is involved in multiple correlations. Still, they provide a basis for our scores, which were tested experimentally and shown to work well in practice. We note that similar assumptions were used previously for other types of analysis of gene expression, with some theoretical and empirical justification, leading to good results [9]. In the discussion we address relaxation of the assumptions.

All class-specific parameters 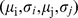 are estimated directly from the input data. They are used to calculate the normalized score, which we call the T-score (or the pairwise DC score) of u and v:
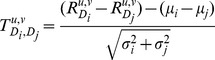
Multiclass data sets are evaluated using “one vs. all” analysis to assign T-scores. For each pair of genes u and v and class 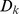, the T-scores 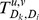 between 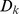 and each other class 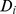 are checked to see if they have the same sign. If the sign is consistent for all classes 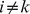 then the aggregated T-score is defined as:
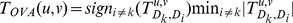
If the signs are inconsistent, however, the score is set to zero. Under this definition, positive aggregated scores mark the cases in which the correlation within 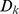 is higher than within all other classes; we call this situation “up correlation.” Negative aggregated scores indicate lower correlation of the pair within 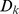. We call this situation “down correlation.” A score of zero is obtained when the DC of u and v is not consistent when 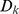 is compared to the other classes.

### The probabilistic framework

We adopted the framework of [9]. In the following sections we use this framework to compare 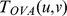 values on real and random data sets, and to compare high and low correlation values within each class. Therefore, we first describe this framework in a general manner. We assume that T-scores belong to one of two distributions with density functions 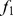 and 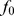, where the probability of belonging to the first distribution is 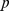, and define the log-likelihood ratio score of a score s as:
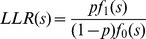
Our analyses assume, for simplicity, that 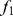 and 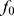 are normal distributions with different means 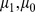 and standard deviations 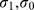. The value 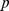 is the prior probability that a score is sampled from 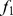. Hence a score 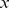 can be transformed into an LLR score:
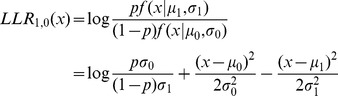
Other distributions can be used as well.

Define 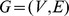 to be a weighted, undirected graph in which the nodes correspond to genes and edge weights correspond to LLR scores. Given a set of edge scores *C*, we would like to test the following two hypotheses:
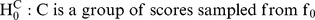


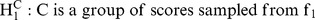
Let 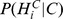 be the posterior probability of 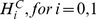. Under a simplifying assumption that the random variables within Care independent, the probability can be rewritten as:
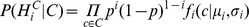
Thus:
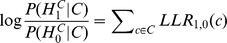
For a set of scores C, we accept 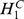 if and only if 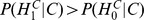. We therefore accept 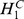 if and only if 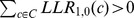.

### Finding DC clusters

DICER compares a class of interest 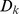 to all other classes by calculating the 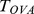 score for each gene pair. We denote the resulting scores as 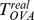. Next, shuffled data sets are created by permuting class labels and are used to calculate 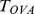 scores. The process is repeated 20 times and the resulting scores are denoted 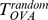. The 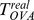 scores are transformed to LLR scores as described above using the distributions of 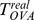 and 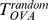. An important parameter in this process is the prior 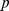 assigned for 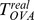. By decreasing this parameter, DICER can control the number of positive LLR scores. Setting a low prior means that fewer pairs of genes will get a positive LLR score. Because this parameter depends on the tested data set we set it as follows:
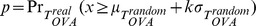
For 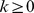 if the distributions of 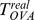 and 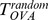 are equal, then by using this prior, almost all LLR scores will be negative.

Note that the 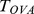 scores do not necessarily follow a normal distribution. Moreover, in multiclass data sets, a large proportion of the gene pairs are scored zero and the distribution is not continuous. Nevertheless, as our experiments show (**Figure 2**), the variance of 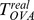 is consistently higher than the variance of 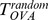, and therefore high T-scores of the real data sets would be assigned with a positive LLR score. In addition, by changing the value of 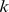 we can control the threshold from which T-scores would be assigned a positive score. Taken together, our LLR score is a flexible approximation of the real LLR score of the underlying data. In our experiments we set a stringent value of 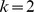.

The gene pairs can be partitioned into those with: (1) non-significant DC (negative LLR score), (2) significant up-correlation (positive T-score, positive LLR), and (3) significant down-correlation (negative T-score, positive LLR). Using these classes we define two weighted, undirected graphs, 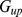 and 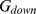, which contain a node for each gene and an edge between each pair of genes. In 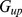, edge weights are defined by keeping the LLR scores (1) and (2), and inverting the sign of the scores in group (3). The idea is that only gene pairs that were significantly up-correlated in 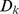 will be assigned positive scores and down-correlated pairs will be penalized. Similarly, in 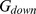, edge weights are defined by keeping the LLR scores (1) and (3), and inverting the sign of the scores in group (2).

DICER uses average-linkage hierarchical clustering [6], [56] to find subgraphs in 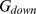 and 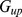. Going from the leaves up, two gene sets are merged as long as the sum of edge scores between them is positive. Sets of size 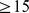 are defined as clusters. The rationale is that these sets correspond to gene clusters that are differentially correlated, as they are more likely to represent a real correlation change than is expected by chance. Setting a size threshold is standard in gene module detection (e.g., a threshold of 15 was used in [9]). In addition, in most cases functionally enriched clusters contained more than 20 genes (66% of the enriched clusters).

Since this part of the algorithm uses sampling, DICER is a non-deterministic algorithm. Hence, different runs might produce different results. We observed that when 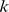 is lower DICER tends to be more stable. For example, for 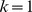, average Jaccard score between repeated runs was larger than 0.85, for all datasets. In this study we have selected a relatively large value of 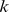 in order to focus on high DC. The stability decreased, but Jaccard score between repeated runs was still above 0.5. Nevertheless, we observed that pathway enrichment analysis was quite stable. For example, ribosome and NDD-related pathways appeared in all different runs in the AD dataset.

### The consistent correlation graph

In this analysis we calculate all gene-pair correlations in every class. We then partition the correlation scores into those with high correlations (denoted “mates”) and those with low correlations (denoted “non-mates”) within each class using the method in [9]. We assume that the distributions of mate and non-mate scores are normal, and use the expectation maximization (EM) algorithm to represent the data as a mixture of two Gaussians. EM evaluates the parameters of the distributions and the prior probability that two randomly chosen elements are mates. Because the distributions may vary between classes, we perform EM for each class separately. We use these parameters to calculate the LLR scores as in the previous section. Denote the LLR score of the gene pair i,j in class c by S(i,j,c). Gene pairs that are co-expressed in all classes will induce a positive LLR score in each class; therefore, to ensure that only gene pairs that are consistently co-expressed in all classes will be assigned a positive score, we integrate all EM runs by assigning to each gene pair the minimal LLR value, namely, S(i.j) = min_c_S(i,j,c). DICER uses these scores as weights of edges in the undirected graph of consistent correlations, denoted as 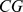.

### Finding meta-modules

In the final phase of the algorithm, DICER uses the graphs 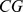 and either 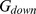 or 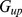 to find meta-modules. We describe the analysis using 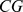 and 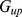; the analysis using 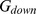 is analogous. We define two disjoint gene sets U and V as *friends* if the sum of edge weights between U and V in 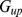 is positive. Based on the probabilistic framework, two modules that are friends are more likely to represent a real correlation change than expected by chance. We define an up-correlated meta-module MM as a pair of non-overlapping gene modules 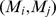 that satisfy: (1) each module 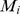 is a sub-graph of 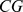 with a positive sum of edge weights, and (2) 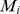 and 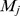 are friends.

Finding the largest meta-module is computationally hard. We proved that there exists no polynomial constant-factor approximation algorithm for finding a maximum size meta-module unless P = NP (See **Text S1**). We therefore developed a heuristic algorithm for meta-module detection. It works in three phases: (1) initial detection of module pairs, (2) greedy merge of pairs, and (3) addition of single genes to a module in a pair. In the next section we describe each phase of the algorithm. See **Figure S5** for an outline.

#### Phase 1: Initial detection of module-pairs

We use a simple local greedy heuristic, akin to [37], to find pairs of initial modules, which we call *seeds*. Using the definition of 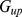, we assume that only a small fraction of the edges will be assigned positive scores. Let 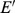 be the set of edges in 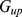 with positive scores, and let 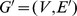 be the unweighted graph induced by 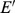. We iteratively select the edge (u,v) in 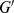 such that u and v together have a maximal number of neighbors. Let 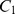 be the set of nodes that are neighbors of u and not neighbors of v, and let 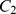 be the set of nodes that are neighbors of v and not neighbors of u. We repeatedly remove nodes from 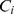 whose sum of edge weights with other nodes in 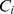 in 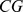 is non positive, or have a non-positive sum of edge weights 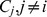, in 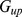.

To determine the order of node removal, we score each node by the sum of scores in 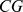 with its set plus the sum of scores with the other set in 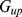. When deciding which node to remove, we consider three candidates: (1) the node that has the minimal score, (2) the node that has the minimal score with the other set in 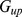, and (3) the node that has the minimal score with its own set in 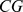. If all three candidates have positive scores we stop and accept the meta-module. We found that in many cases, candidate (1) manifests negative scores both within its group (in 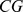) and with the other group (in 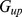). Therefore, in these cases we remove this gene. However, we observed cases in which node (1) has a positive score within its group (in CG) or with the other group (in 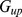), while having a negative score. In these cases we use node (1) as a “guide” for the removal stage: if it has a negative score in 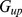 (with the other set) then the edges between the two seeds in 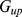 are not heavy enough, and we remove node (2); otherwise we remove node (3). Once a meta-module is detected, its genes are removed and the process is repeated.

#### Phase 2: Greedy merge of module pairs

In the second phase we improve the solution by merging meta-modules. Let 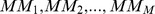 be the current set of meta-modules discovered in phase 1, where each meta-module 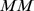 is a pair of non-overlapping gene modules. A pair of meta-modules 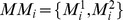 and 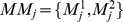 can be merged if one of the merging options 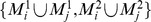 or 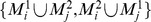 leads to a gain in the scores both within the modules and between them. We iteratively merge the best gain meta-module pair until the gain is negative.

#### Phase 3: Adding single genes to meta-modules

In the third phase we improve the solution by adding to meta-modules single genes that do not belong to any module. A gene 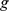 can be added to a meta-module 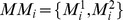 if one of the merging options 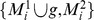 or 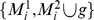 leads to a gain in the scores within the modules and between them. We iteratively look for the best gene and meta-module gain, and add the gene to the appropriate module. We stop this process when the best gain is negative.

### Data sets and preprocessing

Gene-expression profiles from five studies were obtained from GEO [2] using the series matrix of each data set; see **Table 1** for details. To reduce noise and focus on genes that vary across the study, in each data set we used the 3000 probes showing maximum variation and then merged probes by mapping them to Entrez IDs.

### Finding DC gene modules using DiffCoEx

We used the R implementation of DiffCoEx with default parameters.

### Enrichment analysis of pathways and protein complexes

We performed KEGG [57] and protein complex enrichment analysis of gene sets by calculating hypergeometric p-values and false discovery rate (FDR) correction for multiple testing [58]. The background set for the hypergeometric test was the filtered set of genes in the data set. Human protein complex annotations were extracted using BioMart [59], [60]. In total, 237 KEGG pathways and 964 complexes were used.

Because many KEGG pathways have large overlaps with other pathways, after performing the initial enrichment analysis, we filtered the results to remove redundancies. For every cluster, we checked every pair of enriched terms; if the Jaccard coefficient between the gene sets of these terms in the tested cluster was above 0.5, we kept the term with the lower p-value.

### Enrichment analysis of miRNA families

We used the FAME algorithm [38] to test for enrichment of miRNA families in gene sets. We used 2000 sampling steps for evaluating enrichment p-values and use the FDR method to correct for multiple testing (q<0.05).

### Enrichment analysis of known disease and miRNA associations

Having detected a set of miRNA families using the data set of a disease, we tested its enrichment for miRNAs known to be associated with the disease, as recorded in mir2disease [39]. We first converted the associations in mir2disease from MirBase IDs [61] to miRNA families. We then calculated the hypergeometric p-value for the overlap between the detected set and the set of miRNA families associated with the disease. The background set for this test was all miRNA families that had at least one gene target in the data set.

## Supporting Information

Figure S1 The class specific correlation matrices of the first simulated data set. One DC cluster and one meta-module are indicated by blue rectangles.
(TIF)

Figure S2 The class specific correlation matrices of the second simulated data set. The DC cluster and the meta-module are indicated by blue rectangles.
(TIF)

Figure S3 One of the two false DC clusters identified on the second dataset.
(TIF)

Figure S4 miRNA target enrichment in gene sets detected by DICER. For every dataset we used the FAME algorithm to test for enrichment of targets of miRNA families in the gene sets generated by DICER. These included gene clusters, meta-modules and modules (the subgroups of meta-modules). P-values were corrected for multiple testing (0.05 FDR). Because the modules are subgroups of meta-modules, we also calculated the intersection between enriched miRNA families in meta-modules and modules. Note that all data sets except AD are on the same scale.
(TIF)

Figure S5 Overview of the steps of the meta-module discovery algorithm. The seeds that will form the basis for modules of a meta-module are encircled with dashed lines in a-c. Black edges correspond to differentially correlated gene pairs. Red edges correspond to consistently correlated gene pairs. (A) The construction starts from the edge between the yellow nodes. A seed is formed around each of them, containing a set of consistently correlated genes, whereas edges between the two seeds correspond to differentially correlated genes. Genes that are consistently correlated with one of the seeds but are not differentially correlated with the other are excluded. Genes that are differentially correlated with one seed but are not consistently correlated with the other are removed as well. (B) Merging two meta-modules. The resulting meta-module has high differential correlation between the two sides and high consistent correlation within each side. (C) Addition of a single gene to a meta-module. The gene colored green is added to seed2 because it is differentially correlated with seed1 and consistently correlated with seed2. (D) The final meta-module. The two sub-groups of the meta-module are denoted as modules.
(TIF)

Table S1 DiffCoEx and DICER meta-module statistics.
(DOCX)

Table S2 miRNA enrichment analysis results on DICER gene sets discovered in the AD dataset.
(XLSX)

Table S3 miRNA enrichment analysis results on DICER gene sets discovered in the NDD dataset.
(XLSX)

Table S4 miRNA enrichment analysis results on DICER gene sets discovered in the lung cancer dataset.
(XLSX)

Table S5 miRNA enrichment analysis results on DICER gene sets discovered in the SLE dataset.
(XLSX)

Table S6 miRNA enrichment analysis results on DICER gene sets discovered in the IBD dataset.
(XLSX)

Text S1 A proof of the hardness of approximation for the problem of finding a maximal meta-module.
(DOCX)

Text S2 DICER performance on simulated data.
(DOCX)

Text S3 Gene sets discovered in this study.
(ZIP)
