# Supplementary figures and images for: Dissection of Regulatory Networks that Are Altered in Disease via Differential Co-expression
Source: PLoS Comput Biol. 2013 Mar 7;9(3):e1002955. doi: 10.1371/journal.pcbi.1002955 (PMC3591264; doi:10.1371/journal.pcbi.1002955)

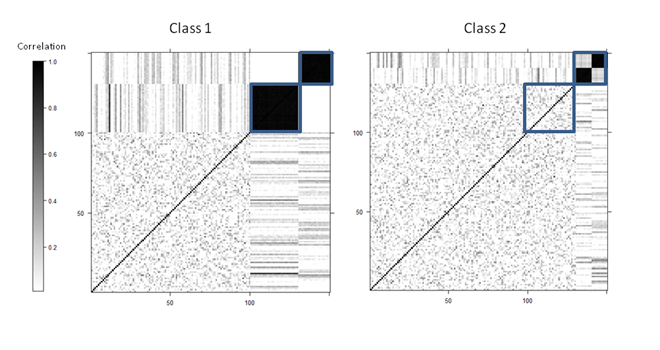

Supplement: Figure S1 — The class specific correlation matrices of the first simulated data set. One DC cluster and one meta-module are indicated by blue rectangles. (TIF) [file pcbi.1002955.s001.tif]

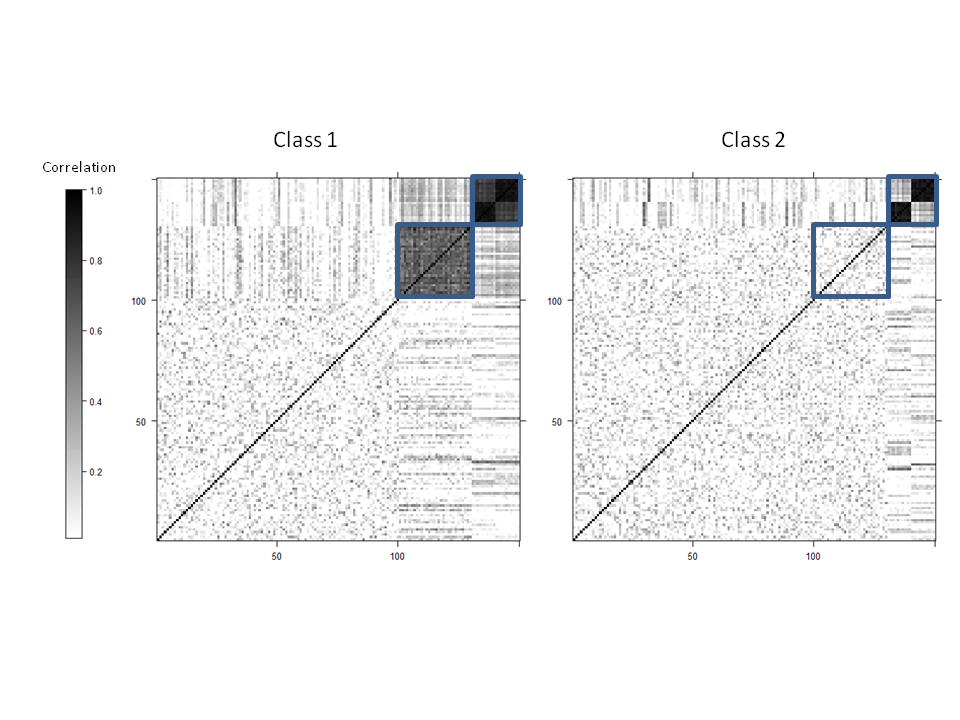

Supplement: Figure S2 — The class specific correlation matrices of the second simulated data set. The DC cluster and the meta-module are indicated by blue rectangles. (TIF) [file pcbi.1002955.s002.tif]

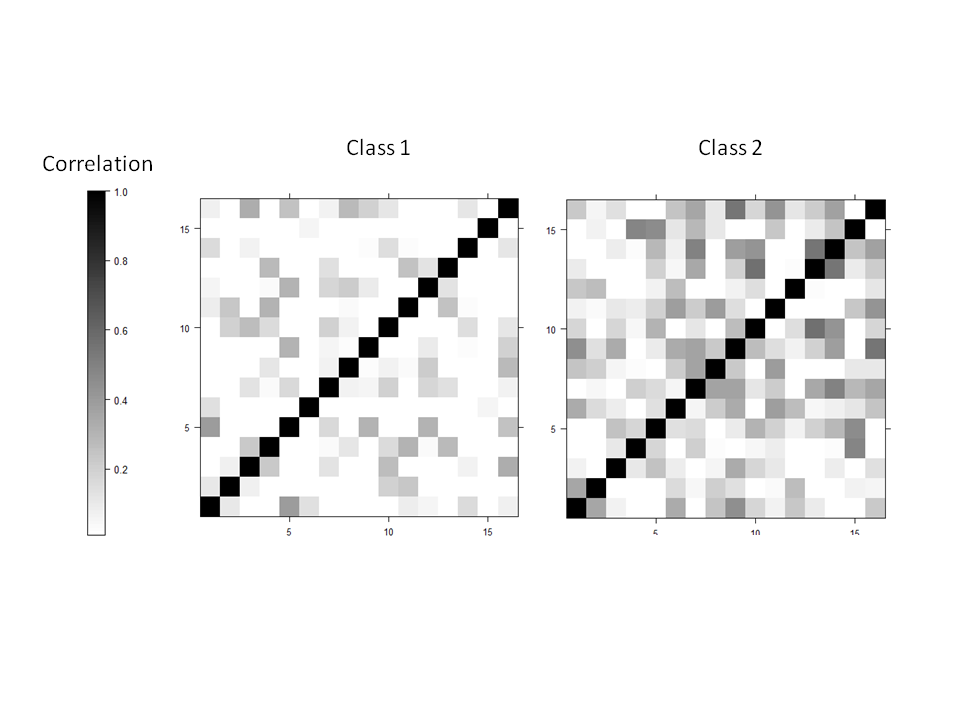

Supplement: Figure S3 — One of the two false DC clusters identified on the second dataset. (TIF) [file pcbi.1002955.s003.tif]

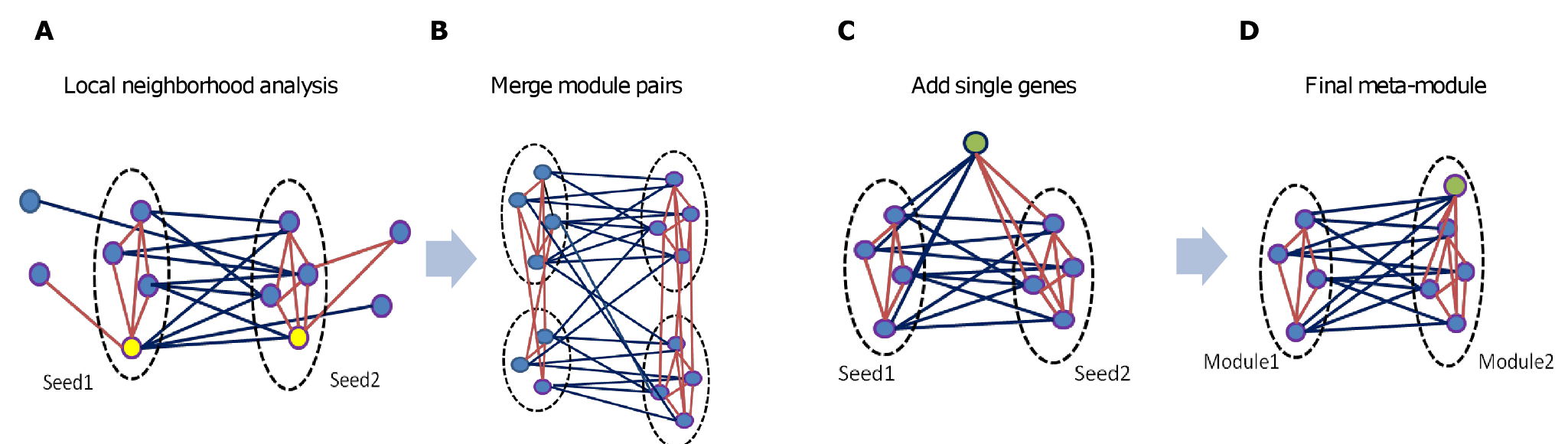

Supplement: Figure S4 — miRNA target enrichment in gene sets detected by DICER. For every dataset we used the FAME algorithm to test for enrichment of targets of miRNA families in the gene sets generated by DICER. These included gene clusters, meta-modules and modules (the subgroups of meta-modules). P-values were corrected for multiple testing (0.05 FDR). Because the modules are subgroups of meta-modules, we also calculated the intersection between enriched miRNA families in meta-modules and modules. Note that all data sets except AD are on the same scale. (TIF) [file pcbi.1002955.s004.tif]

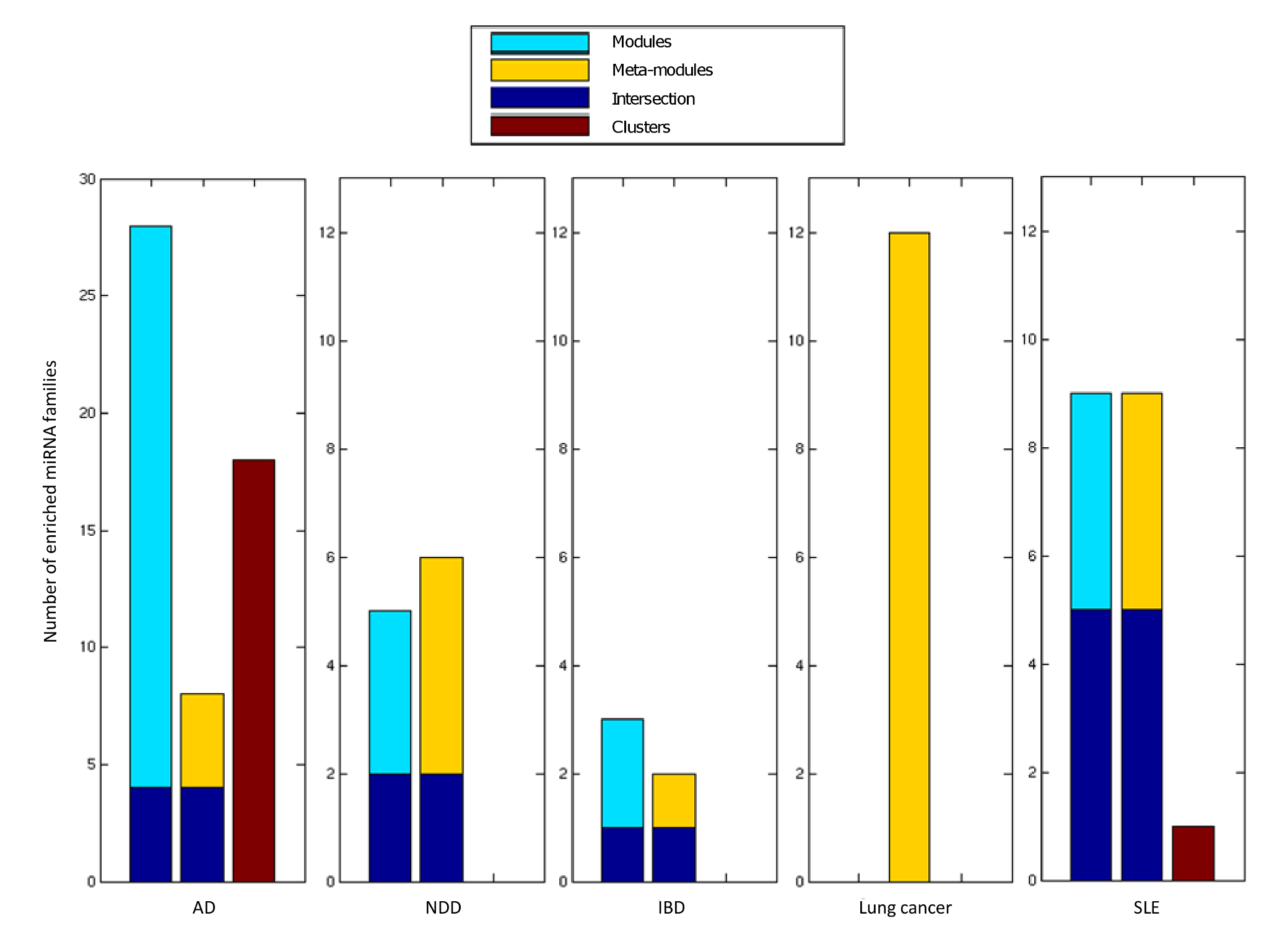

Supplement: Figure S5 — Overview of the steps of the meta-module discovery algorithm. The seeds that will form the basis for modules of a meta-module are encircled with dashed lines in a-c. Black edges correspond to differentially correlated gene pairs. Red edges correspond to consistently correlated gene pairs. (A) The construction starts from the edge between the yellow nodes. A seed is formed around each of them, containing a set of consistently correlated genes, whereas edges between the two seeds correspond to differentially correlated genes. Genes that are consistently correlated with one of the seeds but are not differentially correlated with the other are excluded. Genes that are differentially correlated with one seed but are not consistently correlated with the other are removed as well. (B) Merging two meta-modules. The resulting meta-module has high differential correlation between the two sides and high consistent correlation within each side. (C) Addition of a single gene to a meta-module. The gene colored green is added to seed2 because it is differentially correlated with seed1 and consistently correlated with seed2. (D) The final meta-module. The two sub-groups of the meta-module are denoted as modules. (TIF) [file pcbi.1002955.s005.tif]
