# Supplementary material for: Dissection of Regulatory Networks that Are Altered in Disease via Differential Co-expression
Source: PLoS Comput Biol. 2013 Mar 7;9(3):e1002955. doi: 10.1371/journal.pcbi.1002955 (PMC3591264; doi:10.1371/journal.pcbi.1002955)
Supplement: Table S1 — DiffCoEx and DICER meta-module statistics. (DOCX) [file pcbi.1002955.s006.docx]

**Table S1**: DiffCoEx and DICER meta-module statistics.

| Dataset | DiffCoEx meta-modules | | DICER meta-modules | |
| --- | --- | --- | --- | --- |
|  | Number | Average size | Number | Average size |
| AD | 3 | 48.66 | 50 | 60.1 |
| NDD | 5 | 80.2 | 16 | 37.15 |
| IBD | 2 | 36.5 | 3 | 35.66 |
| Lung cancer | 2 | 278.5 | 20 | 69.2 |
| SLE | 9 | 232 | 24 | 53.16 |
