# Supplementary material for: Dissection of Regulatory Networks that Are Altered in Disease via Differential Co-expression
Source: PLoS Comput Biol. 2013 Mar 7;9(3):e1002955. doi: 10.1371/journal.pcbi.1002955 (PMC3591264; doi:10.1371/journal.pcbi.1002955)
Supplement: Text S1 — A proof of the hardness of approximation for the problem of finding a maximal meta-module. (DOCX) [file pcbi.1002955.s012.docx]

**Text S1: maximal meta-module hardness of approximation**

We show here the hardness of the problem of finding a maximal meta-module.

The formal statement of the problem is as follows: we are given two edge-weighted graphs G=(V,E) and G'=(V,E') with the same vertex set V. A meta-module is two disjoint vertex sets S, T in V such that the sum of edge weights of S and T in G is positive and the sum of weights between S and T is positive in G'. The goal is to find a meta-module of maximum cardinality $|S\cup T|$.

**Theorem:** Finding a maximum size meta-module is NP-hard to approximate within any constant factor.

**Proof:** We show that the problem is NP-hard to approximate within a constant factor via a gap preserving reduction from the max-clique problem . Given the input graph G'' = (V'',E'') with *n* nodes for the maximum clique problem, we define the node set for G and G' as ${V=V}_{1} \cup V_{2}$where $V_{1}$ and $V_{2}$ are copies of V''. For every edge ($u,v$) in E'', let $u_{1}, u_{2}$ and $v_{1}, v_{2}$ be the copies of $u$ and $v$ respectively. In G we set $w\left( u_{1},v_{1} \right)=1$ and $w\left( u_{2},v_{2} \right)=1$. In G' we set $w\left( u_{2},v_{1} \right)=1$, $w\left( u_{1},v_{2} \right)=1$, $w\left( u_{1},u_{2} \right)=1$, and $w\left( v_{2},v_{1} \right)=1$. All other edges are scored -${4n}^{2}$. The reduction is clearly polynomial.

Note first that any module in a meta-module cannot contain a negative edge, since the sum of the weights in such a module would be negative. Hence, every module must correspond to a clique in G''. If there is a clique C with at least b nodes in G'', then it will induce a meta-module with at least 2b nodes in G and G', by taking the two copies of C in G and G' as the modules. If a meta-module with at least 2k nodes exists in G and G', then one of its modules has at least k nodes, and such a module corresponds to a clique in G''. In other words, if there is no clique of size a in G'' then there is no meta-module of size 2a in G and G'. Thus, we have shown a gap preserving reduction:

$$Max clique\left[ a,b \right]\leq_{p} Max meta-module \left[ 2a,2b \right]$$

Since the max-clique problem is NP-hard to approximate within a constant factor [[105-107](#_ENREF_105)], we conclude that the maximal meta-module detection is also NP-hard to approximate within a constant factor.$∎$
