# Supplementary material for: Dissection of Regulatory Networks that Are Altered in Disease via Differential Co-expression
Source: PLoS Comput Biol. 2013 Mar 7;9(3):e1002955. doi: 10.1371/journal.pcbi.1002955 (PMC3591264; doi:10.1371/journal.pcbi.1002955)
Supplement: Text S2 — DICER performance on simulated data. (DOCX) [file pcbi.1002955.s013.docx]

**Text S2: DICER performance on simulated data**

We created simulated expression data and tested DICER’s performance on it. The data contained 150 genes and 60 conditions divided into two classes of 30 conditions each. In these data we planted a DC cluster of 30 genes and a 20-gene meta-module consisting of two 10-gene modules.

To explain the simulation process, we define a *random-pattern* as a vector in which each coordinate is independently sampled from the uniform discrete distribution U(-T,T). T=10 was used as a default. Adding a random-pattern $r$ to a gene expression pattern $g$ of the same size creates a new expression pattern $r+g$. The first simulated data set was created as follows:

1. Create an initial matrix of size 150X60 with values sampled i.i.d. from a standard normal distribution; rows correspond to genes, and columns to conditions, where the first 30 conditions constitute class 1 and the rest are in class 2.
2. Select three disjoint sets of genes $c_{1}, m_{1}, m_{2}$of sizes 30, 10 and 10, respectively.
3. To construct the DC cluster, create a random-pattern $r_{0}$of size 30 and add it to the pattern of the $c_{1}$genes in class 1; Hence, $c_{1}$ patterns will be correlated in class 1 but not in class 2, thereby manifesting DC.
   1. Create three random patterns of size 30, $r_{1},r_{2},r_{3}$;
   2. add $r_{1}$ to the patterns of the genes in $m_{1}$and in $m_{2}$ in class 1;
   3. add $r_{2}$ to the patterns of $m_{1}$ genes in class 2;
   4. add $r_{3}$ to the patterns of $m_{2}$ genes in class 2;

b and c together guarantee that $m_{1}$genes are correlated in both classes, b and d guarantee that $m_{2}$genes are correlated in both classes. b makes sure $m_{1}$and $m_{2}$ are correlated in class 1, and c and d together make sure they are not correlated in class 2.

The two class-specific correlation matrices of this dataset are shown in **Figure S1**. DICER perfectly recovered the cluster and modules in these data: its output contained the DC cluster $c_{1}$and the two modules $m_{1}, m_{2}$reported as one meta-module. No other results were reported.

We also created a second simulated dataset with a weaker DC signal. The data were created similarly to the above, with the following changes:

1. A value of T=2 was used in the random-pattern $r_{0}$. As a result, the correlations are much weaker and closer to the background level and the DC signal is weaker.
2. In creating the meta-module, we changed step (4)a to use a value of T=2 in the random-pattern $r_{1}, r_{2}, r_{3}.$ This weakens the correlative signal in both classes. We also changed step (4)b to: add $r_{1}$ to the patterns of $m_{1}$ in class 1, and add $r_{1}+2*r^{'}$ to the patterns of the genes in $m_{2}$ in class 1, wher$e r^{'}$ is a vector of random i.i.d. s$amples$from a s$tandard normal$distribution. This weakens the co$rrelati$on between $m_{1}$a$nd m_{2}$in class 1.

The two class-specific correlation matrices of this dataset are shown in **Figure S2**. $Since t$he $DC sign$al is weaker here, we changed K to 0.7. Again, DICER perfectly recovered the cluster and modules in these data: its output contained the DC cluster $c_{1}$and the two modules $m_{1}, m_{2}$reported as one meta-module. DICER also reported three additional clusters. The first contained genes, including all the genes of $m_{1}$ and $m_{2}$. The other two consisted of genes that are not in $c_{1}, m_{1}$ or $m_{2}$ , both containing less than 20 genes. See **Figure S3**. However, while the average LLR score of the original cluster $c_{1}$was > 0.8, the average LLR scores of the three other clusters was < 0.2, a 4-fold difference. Even when running DICER with lower K values of 0.6 and 0.5, which adds more noise, the cluster $c_{1}$ and the meta-module $m_{1}, m_{2}$ are discovered.
